# Congenital Long QT Syndrome Presenting as Ventricular Fibrillation and Syncope in a Drug-Naive Elderly Female Patient Without Prior Cardiac History

**DOI:** 10.7759/cureus.88538

**Published:** 2025-07-22

**Authors:** Muhammad Usman Khalid, Safia W Khan, Abdalla Khalil

**Affiliations:** 1 Cardiology, King's College Hospital, NHS Foundation Trust, London, GBR; 2 Medicine, Northwick Park Hospital, London North West University Healthcare NHS Foundation Trust, London, GBR; 3 Acute Medicine, Northwick Park Hospital, London North West University Healthcare NHS Foundation Trust, London, GBR

**Keywords:** acquired long qt, congenital long qt syndrome, drug-induced long qt syndrome, long qt therapy, recurrent syncope

## Abstract

Long QT syndrome (LQTS) is a rare cardiac electrophysiological disorder that predisposes individuals to life-threatening arrhythmias such as torsades de pointes and ventricular fibrillation (VF).

It may be congenital or acquired, with many cases triggered by specific medications or electrolyte disturbances. However, presentations in elderly individuals without prior cardiac history or drug exposure are exceedingly rare.

This case report highlights a unique case of congenital LQTS presenting with VF and cardiac arrest in a 66-year-old woman. She presented with recurrent dizzy spells accompanied by palpitation, cold sweating, abdominal discomfort, nausea, and vomiting. While under observation in the emergency department, she experienced a sudden loss of consciousness with no palpable carotid pulse. Cardiac monitoring revealed VF, and return of spontaneous circulation was achieved after one minute of cardiopulmonary resuscitation. Electrocardiograms revealed a prolonged QTc interval of 600 ms (calculated by Fridericia's formula) and sinus bradycardia of 49 beats/minute initially before the cardiac arrest.

Investigations, including cardiac enzymes, CT scan of the head, coronary angiography, echocardiography, Holter monitoring, and cardiac MRI, were unremarkable. There was no history of QT-prolonging drug use, electrolyte imbalance, or structural heart disease.

Given the high suspicion of congenital LQTS, the patient underwent implantable cardioverter-defibrillator (ICD) placement and was referred for genetic testing. Her first-degree relatives were advised to undergo cardiac screening. This case underlines the importance of considering congenital LQTS in elderly patients presenting with unexplained syncope or VF, especially in the absence of common risk factors. Early recognition and ICD implantation can be life-saving. Genetic testing and family screening remain key components of management in such cases.

## Introduction

Long QT syndrome (LQTS) is a genetic or acquired cardiac disease characterized by delayed ventricular repolarization, depicted as QT prolongation on electrocardiogram (ECG) [1].

This electrophysiological disturbance creates an arrhythmogenic change that predisposes affected individuals to potentially life-threatening ventricular tachyarrhythmias.

Congenital forms result from mutations affecting cardiac potassium, sodium, or calcium channels, whereas acquired forms are typically associated with the use of drugs, electrolyte disturbances, or structural heart disease [2].

Congenital long QT syndrome results from mutations in the KCNQ1, KCNH2, or SCN5A genes, which affect heart cell electrical currents and delay repolarization, while acquired forms are often caused by medications, heart problems, or metabolic issues that block the same currents [1,2].

The clinical significance of LQTS lies in its association with polymorphic ventricular tachycardia, including torsades de pointes and ventricular fibrillation (VF), which may result in syncope or sudden cardiac death [3].

Congenital LQTS affects approximately 1 in 5,000 to 1 in 2,000 individuals, exhibiting variable expressivity and incomplete penetrance [4]. However, population-based genetic studies suggest that the true prevalence may be even higher, as many cases remain undiagnosed due to mild or asymptomatic clinical presentations [4].

Although often presenting in younger individuals with symptoms like syncope, seizures, or sudden cardiac death, congenital LQTS can remain asymptomatic for life and is rarely diagnosed in the elderly, where it may be easily overlooked without QT-prolonging drugs or electrolyte imbalances [4]. In elderly patients, the diagnosis is particularly challenging as symptoms may be attributed to more common age-related conditions such as vasovagal syncope, cerebrovascular disease, or degenerative neurological disorders.

## Case presentation

A 66-year-old woman presented to the emergency department (ED) with sudden-onset dizziness while preparing for bed. The episodes were brief, recurrent, and associated with nausea, vomiting, palpitation, cold sweats, and vague abdominal discomfort. She described intermittent palpitations and generalized weakness in her legs following these episodes. She denied any relation to physical exertion or stress.

She denied any previous history of cardiovascular disease, seizures, or use of QT-prolonging medications. There was no chest pain, shortness of breath, visual disturbances, or recent infection.

She had a similar episode two weeks earlier and another several months ago, but she had not sought medical attention at the time. She was unable to recall if she had any syncope, and she denied any confusion.

Her past medical history was unremarkable, and there was no family history of sudden cardiac death or inherited cardiac conditions.

**Figure 1:**
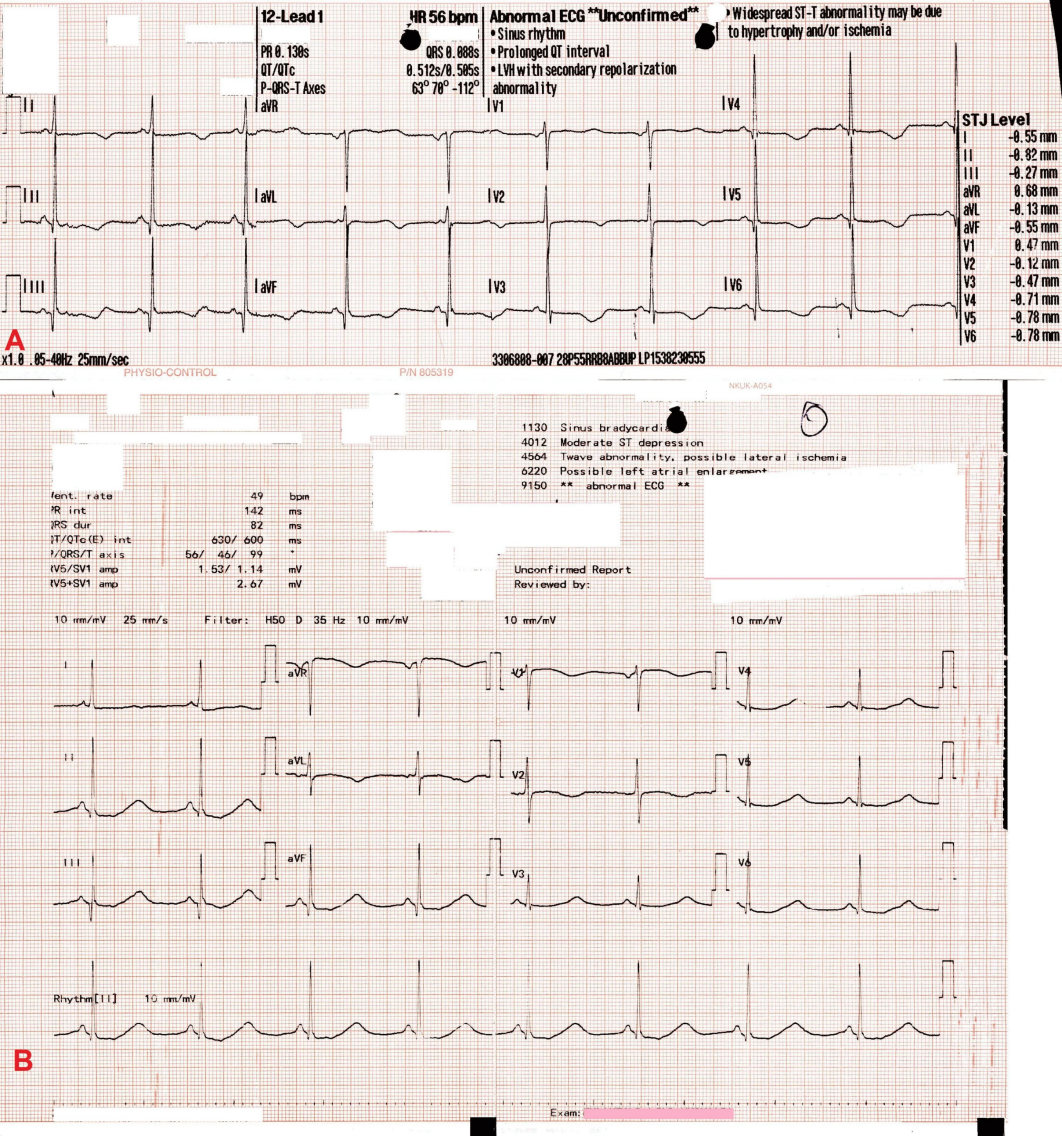
A: ECG performed by paramedics. B: ECG performed in the emergency department. A: ECG (by the ambulance team) revealed sinus bradycardia with a heart rate of 49 beats/minute, a prolonged QTc interval of 580 ms (Fridericia's formula), and an inverted T wave in the chest leads V2 to V6, lateral lead I, aVL, and inferior leads II, III, and aVF. B: ECG (in the emergency department) revealed sinus bradycardia with a heart rate of 49 beats/minute, a prolonged QTc interval of 600 ms, and an inverted T wave in V1, V2, I, and aVL.

On arrival, she was alert and hemodynamically stable. However, while under observation, she experienced another episode of dizziness accompanied by palpitations. Her ECG in the ED revealed a prolonged QTc interval of 600 ms (Fridericia's formula calculation), bradycardia at 49 beats/minute, and T-wave inversion in leads V1, V2, I, and aVL (Figure 1).

Moments later, she lost consciousness, and her carotid pulse was absent. Immediate cardiopulmonary resuscitation was initiated. VF was documented on the cardiac monitor. Return of spontaneous circulation (ROSC) was achieved within one minute.

Her blood tests, including full blood count, renal profile, bone profile, magnesium, thyroid function tests, troponin, and beta-natriuretic peptide, were within normal limits.

**Figure 2:**
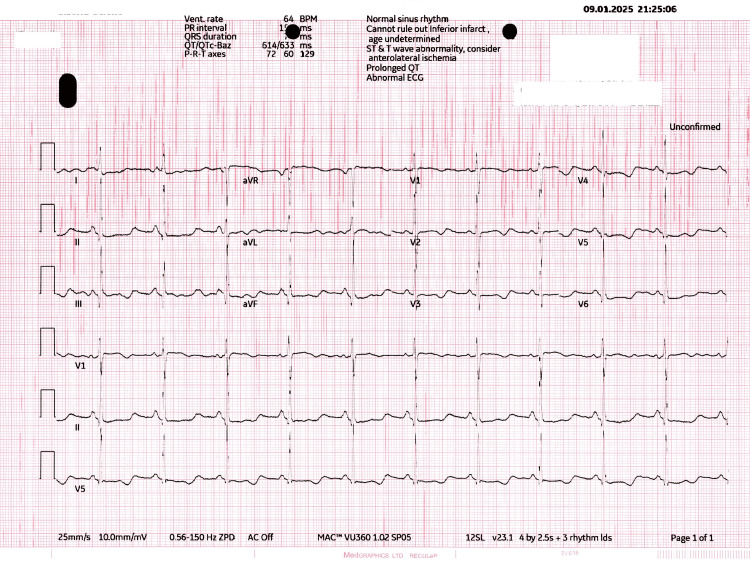
ECG (post-ROSC return of spontaneous circulation) ECG (post-ROSC return of spontaneous circulation) revealed normal sinus rhythm with a heart rate of 63 beats/minute, prolonged QTc interval 630 ms, and inverted T wave in all chest leads, l,aVL, III, and aVF. ROSC: Return of spontaneous circulation

Following resuscitation, her vital signs revealed a blood pressure of 180/100 mmHg and a heart rate of 60 beats per minute. Neurological and cardiovascular examinations were unremarkable. Repeat ECGs showed a prolonged QTc interval of 630 ms post-ROSC, confirming persistent repolarization abnormalities (Figure 2).

**Figure 3:**
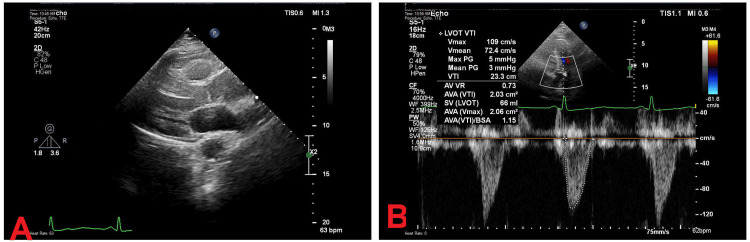
Transthoracic echocardiography. A. Parasternal long-axis view. B. Apical five-chamber view with pulse wave Doppler. A. Transthoracic echocardiogram A: Parasternal long-axis view demonstrating normal morphology and motion of the aortic and mitral valves, with preserved left ventricular wall thickness and contractility. These features are consistent with normal systolic function and absence of significant structural abnormalities. B. Apical five-chamber view with pulsed-wave Doppler evaluation of the left ventricular outflow tract (LVOT), showing a velocity-time integral (VTI) of 23.3 cm, peak velocity of 109 cm/s, and calculated aortic valve area of 2.03 cm². The findings are suggestive of preserved LVOT and aortic valve hemodynamics in this patient.

The patient was stabilized and admitted to the cardiology department for further comprehensive investigations. A CT scan of the head revealed no acute pathology. Echocardiography demonstrated preserved left ventricular function with an ejection fraction of 55-60%, normal valve morphology, and no evidence of structural heart disease or pulmonary hypertension (Figure 3).

**Figure 4:**
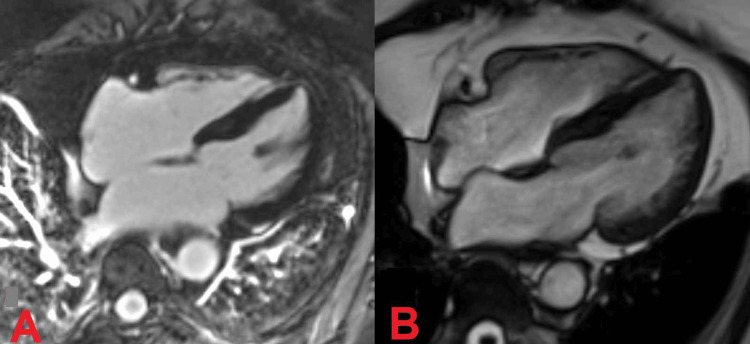
Cardiac magnetic resonance (CMR). A. Four-chamber SSFP image. B. Late gadolinium enhancement (LGE) four-chamber image A. CMR four-chamber SSFP image showing a non-dilated left ventricle with normal wall thickness.  Cine images showed good contractility and a preserved ejection fraction. B. CMR late gadolinium enhancement (LGE) four-chamber image showing a normal LV wall thickness and no hyperintensity, hence the absence of fibrosis, infarction, and infiltration (image was taken 10-20 minutes after gadolinium injection). SSFP: Steady-state free precession

A 24-hour Holter monitor revealed sinus rhythm with isolated supraventricular ectopics and a couplet. Cardiac magnetic resonance showed a non-dilated left ventricle with preserved systolic function and no signs of myocardial fibrosis or infiltration (Figure 4).

Coronary angiography revealed mild coronary artery disease of the left circumflex artery and right coronary artery with no flow-limiting lesion.

Her serum electrolytes, bone profile, and magnesium were within the normal range.

The absence of secondary causes such as electrolyte imbalance, ischemic heart disease, or medication use, alongside ECG findings and clinical presentation, led to a presumptive diagnosis of congenital LQTS. The patient was admitted to the coronary care unit and received an implantable cardioverter-defibrillator (ICD) on day 15 of admission for secondary prevention. Her first-degree relatives were advised to undergo screening for inherited arrhythmias.

She was discharged in stable condition and referred for follow-up at the inherited arrhythmia and pacing clinics.

## Discussion

LQTS is a heterogeneous disorder with a wide spectrum of clinical manifestations, ranging from asymptomatic QT prolongation to sudden cardiac death [4]. Although typically diagnosed in younger individuals, elderly patients may remain asymptomatic for decades, only presenting after a life-threatening arrhythmic event [4]. This case exemplifies such a scenario, where congenital LQTS remained undetected until the patient suffered a VF arrest.

Congenital LQTS is most commonly caused by mutations in genes encoding ion channel subunits, including KCNQ1, KCNH2, and SCN5A [5]. These mutations impair cardiac repolarization, thereby prolonging the QT interval and increasing susceptibility to torsades de pointes and VF [6]. LQTS might not cause any symptoms throughout a person's life, but these can show up under certain conditions, like when someone develops hypokalemia or is taking antipsychotic medications [7].

In our patient, the QTc intervals exceeding 600 ms and the absence of reversible triggers strongly favored a diagnosis of congenital rather than acquired LQTS. The patient was diagnosed with LQTS according to the latest consensus guidelines, which require a combination of clinical factors, including a QT interval of 480 ms or more, relevant symptoms, and a high likelihood based on the Schwartz score [8].

Complete assessment involving echocardiography, coronary angiography, cardiac MRI, detailed medication history, and electrolyte evaluation becomes essential to rule out acquired or secondary causes, and in the absence of any structural or biochemical abnormalities, inherited channelopathies should be considered even in elderly individuals [9].

Our patient's preserved cardiac structure and lack of comorbidities raised the index of suspicion for a congenital disorder.

Management strategies for LQTS aim to prevent sudden cardiac death. Beta-blockers, particularly nadolol and propranolol, are considered first-line in symptomatic patients, though their efficacy is limited in high-risk individuals [10]. In cases of syncope, sustained VT, or cardiac arrest, as in our patient, ICD implantation is strongly recommended [11]. Genetic testing not only aids in confirming the diagnosis but also helps guide management and identify relatives at risk [12].

Interestingly, delayed onset presentations of congenital LQTS, as seen in this case, may be due to incomplete penetrance, modifying genetic or environmental factors, or age-related changes in autonomic tone [12]. While less commonly reported, such cases demand vigilance and a tailored approach to risk stratification.

Our case also emphasizes the importance of family screening. As LQTS follows an autosomal dominant inheritance pattern in most subtypes, early detection in asymptomatic relatives allows timely intervention and potentially prevents fatal arrhythmic events. Genetic counseling and cascade testing remain central to comprehensive LQTS care.

## Conclusions

This case underscores an uncommon presentation of congenital LQTS in an elderly female patient without prior cardiac history or QT-prolonging drug exposure. The significant QTc prolongation, absence of secondary causes, and occurrence of VF support a diagnosis of congenital LQTS.

Timely recognition, effective resuscitation, and ICD implantation were critical in preventing sudden cardiac death. This case highlights the importance of considering congenital aetiologies in older adults presenting with unexplained syncope or arrhythmic events. A comprehensive evaluation, including genetic testing and family screening, plays a vital role in managing LQTS and identifying individuals at risk, thereby enabling preventive strategies and long-term care.
